# Recognizing the Health Benefits of Plant‐Sourced Omega‐3 Stearidonic Acid: Exploring Its Complementary Role to Preformed EPA/DHA

**DOI:** 10.1002/lipd.12452

**Published:** 2025-06-27

**Authors:** Ella J. Baker, Greg Cumberford, Patrick Hanaway

**Affiliations:** ^1^ School of Human Development and Health, Faculty of Medicine University of Southampton Southampton UK; ^2^ NIHR Southampton Biomedical Research Centre University Hospital Southampton NHS Foundation Trust and University of Southampton Southampton UK; ^3^ Natures Crops International Kensington Prince Edward Island Canada; ^4^ Family to Family Weaverville North Carolina USA

**Keywords:** Ahiflower oil, docosahexaenoic acid, eicosapentaenoic acid, omega‐3, stearidonic acid, sustainability

## Abstract

Long‐chain (LC) omega‐3 polyunsaturated fatty acids (PUFAs) like eicosapentaenoic acid (EPA), and docosahexaenoic acid (DHA) are crucial for optimal development, healthy aging, and disease management. Traditionally sourced from fatty fish, these omega‐3 PUFAs face sustainability challenges, prompting increased exploration of plant‐based alternatives, such as stearidonic acid (SDA). Recent studies highlight the efficient conversion of SDA to EPA, meaning that SDA may offer similar health benefits to EPA, including immune, joint, cognitive, and gut microbiome modulation (with distinct SDA‐derived metabolites). This mini‐review explores new research on SDA and its potential to deliver human health benefits. SDA‐rich oils, notably *Buglossoides arvensis* oil (RBO; also known as Ahiflower oil) provide an eco‐friendly, sustainable alternative to fish‐derived omega‐3 PUFAs. As concerns about marine omega‐3 PUFA sources grow, SDA‐rich oils present a viable option for clinicians and consumers seeking effective omega‐3 PUFA supplementation.

**Abbreviations**
ALA: alpha‐linolenic acid
CSIA: compound specific isotope analysis
DHA: docosahexaenoic acid
DPA: docosapentaenoic acid
EPA: eicosapentaenoic acid
ETA: eicosatetraenoic acid
GLA: gamma‐linolenic acid
IL‐10: interleukin 10
LA: linoleic acid
LC‐PUFA: long‐chain polyunsaturated acid
PUFA: polyunsaturated acid
RBC: red blood cell
RBO: refined *Buglossoides arvensis* oil
SDA: stearidonic acid

## 1 Introduction

Long‐chain (LC) omega‐3 polyunsaturated fatty acids (PUFAs), such as eicosapentaenoic acid (EPA) and docosahexaenoic acid (DHA), play a vital role in promoting optimal development, supporting healthy aging, and helping in the management of various health conditions (Troesch et al. 2020; Calder 2018; Jiang et al. 2021). Traditionally obtained from fatty fish, these omega‐3 PUFAs face sustainability concerns (Baker 2023; Salem Jr. and Eggersdorfer 2015; Glencross et al. 2025), driving increased interest in plant‐based alternatives. Alternative sources of EPA and DHA to fish oil include algae, genetically modified canola and camelina, krill, and *Calanus* zooplankton (Baker 2023). However, sources of omega‐3 PUFAs other than EPA and DHA but which have the potential to be converted to EPA and DHA are important to consider (Baker et al. 2016). In this context, a series of recent peer‐reviewed studies have highlighted the anti‐inflammatory, immune‐ and gut‐microbiome modulating, and metabolic effects of stearidonic acid (SDA) (Laevski et al. 2024; Metherel, Klievik, et al. 2024; Roussel et al. 2024; Seidel et al. 2024).

**FIGURE 1:**
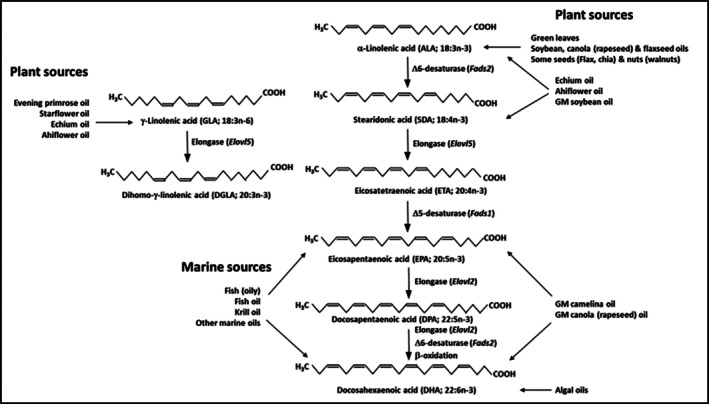
Pathway of conversion of α‐linolenic acid to docosahexaenoic acid and gamma linolenic acid to dihomo gamma linoelnic acid, showing sites of entry of preformed omega‐3 fatty acids from foods or supplements. Modified from Baker (Baker 2023). ALA, α‐linolenic acid; DHA, docosahexaenoic acid; DPA, docosapentaenoic acid; EPA, eicosapentaenoic acid; ETA, eicosatetraenoic acid; GLA, gamma linolenic acid; SDA, stearidonic acid; Italicized names in parentheses after enzyme names are the genes encoding the protein.

Because humans and other animals cannot produce it, alpha‐linolenic acid (ALA) is a biologically essential omega‐3 PUFA. Humans must obtain ALA primarily from dietary plant sources in order to support optimal cellular function and to form other pivotal omega‐3 PUFAs like EPA and DHA, which can also be ingested directly through oily fish or supplements such as fish, algal or krill oils. ALA is the metabolic precursor to EPA, docosapentaenoic acid (DPA), and DHA. The first step of that pathway is catalyzed by delta‐6 desaturase and produces SDA (Figure 1). ALA is found in abundance in flaxseeds (~57% of fatty acids), chia seeds (~61%), hemp seeds (~20%), and their respective oils as well as in some nuts such as walnuts. To a lesser degree, ALA is also present in canola (rapeseed) oil (approximately 9%–11% of fatty acids) and soya bean oil (about 7%–8%). Among these, only hemp seeds and their oil also contain SDA (Table 1). SDA is found in refined *Buglossoides arvensis* oil (RBO, 17%–21% of fatty acids) and *Echium plantagineum* oil (EO, 11%–13%). The SDA content of other available plant‐based sources is 2%–3% in hemp seed oil and 2%–4% in black currant seed oil. Older studies with EO demonstrated increases in circulating EPA and DPA but not DHA (Miles, Banerjee, and Calder 2004; Miles, Banerjee, Dooper, et al. 2004; Surette et al. 2004; Pieters and Mensink 2014; Dittrich et al. 2014; Kuhnt et al. 2014; Greupner et al. 2019). However, Echium is grown in limited quantities and its cultivation is restricted in many regions due to its status as an invasive species and its toxicity issues with grazing animals (Zhu et al. 2017). *Buglossoides arvensis* is cultivated regeneratively in the United Kingdom, where it has been trademarked as Ahiflower. In addition to ALA and SDA, RBO contains the bioactive omega‐6 PUFA γ‐linolenic acid (6% of fatty acids). Table 1 compares the fatty acid composition of commonly available plant and algal oils.

**TABLE 1:** Typical fatty acid composition of selected plant and algal oils.

Oil source	Total % PUFAs	DHA	EPA (+DPA)	SDA	ALA	GLA	LA	OA
Plant								
*Buglossoides arvensis*	83	0	0	20	45	6	12	10
Camelina (non‐GMO)	55	0	0	0	38	0	17	15
Canola	30	0	0	0	10	0	20	60
Chia	79	0	0	0	61	0	18	8
Echium	63	0	0	12	27	10	14	0
Flaxseed	71	0	0	0	57	0	14	16
Hempseed	75	0	0	2	20	3	50	13
Nutriterra GMO canola	36.5	9	1.5	0	19	0	7	45
Soybean	68	0	0	0	13	0	55	18
Algae								
Schizochytrium species	41	40	1	0	0	0	0	0

*Note*: Data taken from (Surette et al. 2004; Ghazani and Marangoni 2016; Clemente and Cahoon 2009; Imran et al. 2016; Goyal et al. 2014; Kara et al. 2010; Moser 2010; Lin et al. 2022; Lefort et al. 2016; Sijtsma and de Swaaf 2004).

Abbreviations: ALA, alpha‐linolenic acid; DHA, docosahexaenoic acid; EPA, eicosapentaenoic acid; GLA, gamma‐linolenic acid; LA, linoleic acid; OA, oleic acid and SDA, stearidonic acid.

[Corrections made 22 July 2025, after first online publication: The data row for “Calanus (waxy ester)” has been removed from Table 1. The reference citation for Schots et al. 2020 was removed from the list of references in the note section of Table 1. Typos in the the abbreviations section of Table 1 for linoleic and oleic acid have been corrected.]

**FIGURE 2:**
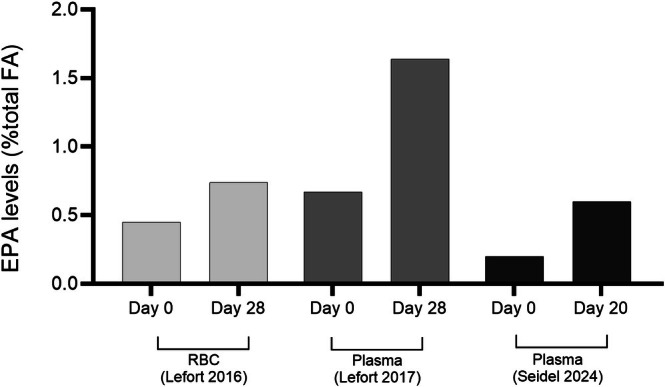
Circulating EPA accrual from *Buglossoides arvensis* oil consumption in humans. Data taken from (Seidel et al. 2024; Lefort et al. 2016, 2017).

Dietary SDA has been shown to raise EPA levels in plasma phospholipids and in red blood cells approximately five times more efficiently than ALA in humans (James et al. 2003). Moreover, SDA has been shown to increase EPA levels in humans at approximately 30%–40% the efficiency of EPA itself, depending on intake levels and sex, with women generally converting both ALA and SDA to EPA more efficiently than men (James et al. 2003; Krul et al. 2012). SDA‐rich RBO increased circulating EPA levels significantly (+64% in red blood cells and + 150% in plasma) in healthy adults over the course of 28 days, contributing positively to the Omega‐3 Index, although there was no change in blood DHA levels (Lefort et al. 2016). The most recent human dietary intervention trial, a three‐way randomized crossover trial in healthy young males aged 18–31 years, reported that RBO intake at 9.1 g/day tripled circulating plasma EPA levels (+200%) in 20 days (Figure 2) (Seidel et al. 2024). Likewise, human dietary intervention trials with EO have demonstrated circulating EPA and DPA accrual (but not DHA) (Kuhnt et al. 2014, 2016). These trials are consistent with findings from earlier intervention trials using genetically modified soya bean oil containing SDA at levels comparable to RBO (Harris et al. 2008; Lemke et al. 2010).

SDA has been recognized for more than 30 years as a notable omega‐3 PUFA having anti‐inflammatory, digestive, and pro‐collagen forming effects, and bypassing the liver's rate‐limited enzymatic process of forming EPA from ALA (Surette 2013; Walker et al. 2013). New evidence in mice suggests that ALA and SDA can also act as substrates for the biosynthesis of DHA in key tissues like the liver, adipose, and brain with comparable DHA turnover efficiency as from pure marine DHA at isomolar PUFA intake levels (Metherel, Klievik, et al. 2024). Furthermore, recent research provides further support for the role of SDA in maintaining immune, joint, and cognitive health, and gut microbiome function (Laevski et al. 2024; Roussel et al. 2024; Lucchinetti et al. 2022, 2025).

Recognizing these effects is relevant to practitioners and consumers who might think improved omega‐3 PUFA status and related wellness benefits are only attainable from fish or algal omega‐3 PUFA sources. This is especially important now, as rising ocean temperatures are affecting fish‐based omega‐3 PUFA supply chains, causing omega‐3 fish oil supplement brands to face challenges in securing consistent, high‐quality supplies (Glencross et al. 2025). Interestingly, in comparisons to fish oil in newly published animal models, examining a range of inflammation and metabolism markers and metabolites, SDA‐rich sources, such as RBO, are showing comparable and/or complementary actions as fish oil sources while being sustainable, scalable, and not requiring genetic modification to raise circulating EPA levels.

The latest findings, summarized below, suggest that SDA‐rich oils may provide similar health benefits to marine‐derived omega‐3 PUFAs, including supporting cardiovascular, immune, gut–brain axis, digestive, and joint/mobility health, at comparable intake levels to standard fish oil. While the data points to promising potential for SDA‐rich oils, more research is needed, and SDA‐rich oils should be further evaluated in comparative human dietary interventions and prospective cohort trials, specifically looking at inflammation markers, joint health, gut–microbiome modulation, and effects on the gut–brain axis, liver protection, and insulin sensitivity.

### 1.1 Recent Discoveries on the Metabolism of SDA and the Biosynthesis of DHA

Older research supports that SDA is well converted to EPA (James et al. 2003). However, there were doubts about conversion of EPA, and therefore of SDA, to DHA. Studies were mainly based on measuring enrichment of EPA and DHA in blood plasma or serum or blood cells such as red blood cells and are summarized elsewhere (Baker et al. 2016). In more recent years, new ways of measuring the metabolism and interconversion of omega‐3 PUFAs have been developed. These studies use compound‐specific isotope analysis (CSIA) to determine DHA synthesis/turnover. Contrary to previously accepted views which supported supposedly “inefficient” DHA synthesis from plant omega‐3 PUFA sources (Baker et al. 2016), Metherel et al. describe efficient DHA synthesis/turnover in brain and liver tissues in mice fed plant‐based omega‐3 PUFAs (Metherel, Klievik, et al. 2024).

In a 2015 review, authors examined data from both rodent models and healthy young adult males, concluding that DHA synthesized from ALA is likely sufficient to meet the brain's daily DHA requirements, which were estimated to be between 2.4 and 3.8 mg in humans. The review highlighted evidence that animals fed ALA‐only diets showed brain DHA concentrations comparable to those fed preformed DHA (Domenichiello et al. 2015). This suggests that at a relatively low ALA‐to‐DHA conversion rate of 1%, a daily brain requirment of 4 mg of DHA could be met with just 400 mg of dietary ALA, which could easily be provided by 1 g of RBO or 4 g of walnuts.

In a mouse omega‐3 feed‐switching trial, new brain tissue DHA was accrued from dietary RBO, due to DHA synthesis from SDA, with comparable real‐time efficiency as from a pure marine DHA source, even though plasma DHA levels were not changed significantly over 120 days (Metherel, Klievik, et al. 2024). Feeding levels of the dietary oils were isomolar for ~2.7% total PUFAs and indexed to adult human‐realistic intakes for these dietary sources. This new evidence indicates that DHA maintenance needs may be being matched by DHA biosynthesis rates in high‐priority tissues, even though blood or plasma DHA levels are not increasing significantly. This may indicate that typical blood measurements of omega‐3 PUFA status, including the Omega‐3 Index, which provides the percent total of EPA + DHA levels in red blood cells or whole blood, may underestimate DHA biosynthesis and accrual in some tissues such as the brain. Indeed the authors state “This suggests that [RBO] feeding can supply DHA to tissues at similar rates compared to DHA feeding alone, and once again reveals a limitation of considering DHA levels only as a marker of DHA status in tissue” (Metherel, Klievik, et al. 2024). This finding was in contrast to observations with dietary flaxseed oil: mice fed flaxseed oil displayed significantly slower DHA synthesis/turnover kinetics compared to DHA feeding alone. The authors suggest these findings may indicate flaxseed oil's relatively slower ability to supply DHA to tissues and are consistent with older observations of poor conversion of ALA to DHA (Baker et al. 2016). The contrast between RBO and flaxseed oil demonstrates DHA biosynthesis efficiency gains from dietary oils rich in SDA versus the delta‐6 desaturase‐dependent pathway required to metabolize ALA.

Nevertheless, Rotarescu et al. recently reported on DHA synthesis and turnover rates in male and female mice as determined by CSIA (Rotarescu et al. 2024). Using a diet‐switch CSIA model, mice were allocated to one of three 12‐week dietary interventions for which total omega‐3 PUFAs were maintained throughout and the only difference was in the source of omega‐3 PUFAS: ALA, EPA, or DHA. The researchers used: (1) 4‐week low δ13 C‐ALA preswitch diet → 8‐week high δ13 C‐ALA post switch diet; (2) 4‐week low δ13 C‐EPA preswitch diet → 8‐week high δ13 C‐EPA postswitch diet; or (3) 4‐week low δ13 C‐DHA preswitch diet → 8‐week high δ13 C‐DHA postswitch diet. They reported that ALA‐to‐DHA synthesis rates in the whole body in mice are as much as 47 times higher than estimated from measurements made in circulating serum alone and, further, that higher DHA intakes inhibit natural dietary ALA → EPA → DHA liver synthesis pathways for making and storing DHA (Rotarescu et al. 2024). More recent findings indicate that dietary intake of preformed DHA significantly suppresses endogenous DHA synthesis via downregulation of hepatic elongation of very long‐chain fatty acids protein 2 (ELOVL2) expression (Metherel, Valenzuela, et al. 2024). This suppression was shown to occur at both the transcriptional and translational levels, resulting in reduced ELOVL2 mRNA and protein abundance. Notably, the observed effect was comparable to the phenotype of ELOVL2 knockout mice, where DHA biosynthesis is impaired due to the absence of ELOVL2. Additionally, concentrations of key biosynthetic intermediates, including DPA, were markedly decreased, further supporting the conclusion that exogenous DHA induces a feedback inhibition of its own synthesis (Metherel, Valenzuela, et al. 2024).

These recent findings suggest that the common claim regarding the “inefficiency” of human (and other mammalian) conversion of plant‐based omega‐3 PUFAs to DHA (often supported by focusing solely on changes in DHA levels in plasma, serum, or circulating blood cells) may overlook the effectiveness with which other tissues naturally synthesize and utilize DHA from adequate intakes of plant‐based omega‐3 precursors, especially in the absence of preformed DHA in the diet. Burdge has suggested that plant‐based diets that exclude EPA and DHA do not appear to be detrimental to health (Burdge 2022). He also suggested that EPA and DHA synthesis [is] a product of human evolutionary history; in this context, hepatic capacity for ALA conversion, accompanied by PUFA synthesis in peripheral tissues and metabolic adaptations in response to physiological increases in demands, can be regarded as appropriate for meeting human EPA and DHA requirements (Burdge 2022). This may also be relevant when considering rich sources of ALA and SDA. Once DHA is synthesized, it can be stored in triglycerides within adipose tissue, which serves as a key reservoir supporting overall body DHA status. However, this is not reflected in the Omega‐3 Index, which primarily measures DHA and EPA levels in the phospholipids of red blood cell membranes. Given that dietary SDA sources lead to more efficient EPA accumulation in circulating cells compared to ALA‐only sources, along with improved DHA turnover rates in key tissues such as the liver and brain, these findings further emphasize the need to investigate not just conversion efficiencies but also the physiological outcomes of dietary SDA compared to preformed EPA/DHA sources.

### 1.2 Recent Discoveries on the Anti‐Inflammatory Gut‐ and Immune‐Supporting Actions of SDA

Sung et al. examined the effects of SDA and DHA in bacterial lipopolysaccharide (LPS)‐stimulated RAW264.7 macrophages (Sung et al. 2017). They found that pretreatment with SDA or DHA (100 μM) suppressed inducible nitric oxide synthetase‐mediated nitric oxide production in response to LPS. Furthermore, incubation with SDA or DHA was shown to downregulate NF‐κB activity (nuclear translocation) and inactivate MAPK. They indicated SDA may be as effective as DHA against inflammatory responses in LPS‐stimulated macrophages and may be seen as an alternative to fish‐derived omega‐3 PUFAs for preventing or treating inflammatory disease (Sung et al. 2017).

Dietary SDA is also now recognized to facilitate upregulation of anti‐inflammatory interleukin‐10 (IL‐10) in human macrophages (Lefort et al. 2017). IL‐10 is a cytokine that is part of the body's natural inflammation and resolution response, notably helping the body control excess inflammation, which may be due to many factors including chronic exposure to inflammatory stimuli, seasonal immune challenge, intensive exercise, and aging. A human trial by Lefort et al. examined the effects of various doses of RBO (2.7–9.1 g/day, dose‐ranging) in healthy individuals for 28 days (Lefort et al. 2017). They described enhanced production of IL‐10 in LPS‐stimulated whole blood of subjects receiving the highest dose of RBO (Lefort et al. 2017). The authors wrote, “It is noteworthy that the abundance of IL‐10 can predict the severity of several human diseases with an inflammatory etiology, with low circulating IL‐10 suggesting a greater disease severity … [RBO] may share immune‐modulating properties that are typically associated with the consumption of marine oils” (Lefort et al. 2017).

Additionally, in a trial involving total parenteral nutrition (TPN) in mice, an RBO‐dominant lipid emulsion resulted in systemic upregulation of IL‐10. Intravenous infusion of a lipid emulsion containing RBO, olive oil, and coconut oil (50:25:25 vol/vol/vol) tested in C57Bl6 mice for 7 days demonstrated higher liver and muscle IL‐10 levels, and an improved IL‐10 to interleukin 6 (IL‐6) ratio compared to soybean oil and fish oil emulsions. Furthermore, the RBO‐dominant emulsion was seen to confer significant immune support, insulin regulation, and gut microbiome balancing effects in marked contrast to fish oil‐ and soybean oil‐based emulsions used at the same level (Lucchinetti et al. 2022). Upregulating IL‐10 may also facilitate hormonal balance and regulate maternal immunity in ways that confer recognized benefits for women's reproductive fertility (Thaxton and Sharma 2010). Furthermore, more recent findings demonstrated superior anti‐inflammatory outcomes in neonatal piglets administered an RBO‐based emulsion (Lucchinetti et al. 2025). Piglets received the lipid emulsion composed of RBO, olive, and coconut oils (Vegaven) via TPN for 2 weeks, compared to a conventional lipid emulsion blend of soybean, coconut, olive, and fish oils (SMOFlipid). The study reported significantly reduced inflammation in the liver, pancreas, and brain in piglets treated with Vegaven compared to SMOFlipid (Lucchinetti et al. 2025). They reported a significant reduction in the concentrations of endotoxin (*p* = 0.004), IL‐6 (*p* = 0.003), and TNF‐α (*p* = 0.017) in the brain tissues of Vegaven‐treated piglets compared to those treated with SMOFlipid. Similarly, Vegaven‐treated piglets exhibited significantly lower levels of endotoxin (*p* < 0.001), TNF‐α (*p* = 0.005), and IL‐6 (*p* = 0.031) in pancreatic tissue, along with a significant increase in IL‐10 (*p* = 0.011) concentrations. A similar pattern was observed in the liver, where Vegaven treatment resulted in decreased levels of IL‐6 and TNF‐α (*p* < 0.05), and a significant elevation in IL‐10 concentrations (Lucchinetti et al. 2025).

Additionally, the RBO‐based emulsion improved gut barrier integrity, enhanced insulin signaling, and showed better glucose regulation (Lucchinetti et al. 2025). Commenting on these findings, Calder said “… the findings suggest anti‐inflammatory, immune supporting, insulin sensitizing, and hepatoprotective effects of the Ahiflower oil emulsion relative to the comparator(s). These effects are all clinically relevant … The superiority of the Ahiflower oil‐based emulsion over those containing fish oil is quite remarkable and requires explanation” (Calder 2025).

Another recently published animal trial found that RBO performed comparably to fish oil in reducing symptoms of rheumatoid arthritis when the oils were given at the same daily intakes equivalent to ~6 g/day in adult humans (Laevski et al. 2024). C57Bl6 mice were fed a control diet (western diet), low RBO, high RBO, or fish oil for 5 weeks, following which rheumatoid arthritis was induced using the K/BxN model. The impact of these diets on platelets, platelet microvesicles (PMVs), and inflammatory markers, such as clinical index, ankle thickness, and cytokine/chemokine release, was assessed. There was a significant decrease in PMV production in mice consuming either low RBO or fish oil diets compared to mice consuming the control diet. Referring to decreased ankle swelling, increased mobility, and suppression of pro‐inflammatory cytokine MIP‐1α, and chemokine CXCL5, the authors summarized, “Low concentrations of dietary *B. arvensis* oil may have similar anti‐inflammatory potential seen with dietary fish oil supplementation” (Laevski et al. 2024).

Fewer studies have examined the effects of SDA on oxylipins. Oxylipins are oxygenated metabolites of omega‐3 and omega‐6 fatty acids (prostaglandins, thromboxanes, leukotrienes, etc.), some of which have well‐recognized anti‐inflammatory and pro‐inflammatory activities (Chiang and Serhan 2020; Calder 2020). These metabolites greatly affect responses to injury, immune challenge, and stress.

One study measured changes in circulating oxylipins in humans and cultured liver cells (Seidel et al. 2024). The researchers found that RBO efficiently increases plasma EPA and corresponding EPA‐derived oxylipins (hydroxy and dihydroxy‐EPA metabolites [5‐, 12‐, 15‐HEPE, 8,9‐DiHETE, 11,12‐DiHETE, 14,15‐DiHETE, 17,18‐DiHETE]) while lowering arachidonic acid‐derived oxylipins (12‐, 15‐HETE) and PGF2α. They suggested RBO caused a “distinct shift in the entire oxylipin pattern” (Seidel et al. 2024).

Finally, potential effects of RBO on the human gut microbiome have been published recently, using a simulated human gut model referred to as the Mucosal Simulator of the Human Intestinal Microbial Ecosystem (M‐SHIME) (Roussel et al. 2024). RBO provided to the simulated gut at 1.2 g/day favorably modulated the gut microbiome to a more anti‐inflammatory profile (raising the abundance of *Bacteroides* and lowering that of *Clostridia*). They discovered SDA‐induced *Bacteroides* genera shifts produced stearidonoyl ethanalomine (SDEA) as well as *N*‐acyl‐3‐hydroxy‐palmitoyl glycine (commendamide), a well‐recognized GPR132 agonist associated with gut–brain axis immune modulatory and gut inflammation‐calming effects. They also showed significant upregulation of the short‐chain fatty acid propionate. Compared to conventional fish EPA/DHA sources which contain no SDA, the authors stated, *“*Our findings suggest a more targeted and selective influence of Buglossoides oil, aligning with the criteria accepted to define a food supplement as a prebiotic” (Roussel et al. 2024).

Taken together, these findings consistently support RBO's beneficial anti‐inflammatory actions and support the potential role of RBO as a beneficial dietary component in mitigating the harmful effects of chronic low‐grade inflammation and inflammaging, particularly when combined with other healthy dietary and lifestyle practices.

### 1.3 Epidemiological Considerations Relevant to RBO and SDA

Looking beyond individual controlled intake trials to epidemiological evidence, a recent study using the National Health and Nutrition Examination Survey (NHANES) data which included considerations for SDA's role in the diet, found that higher levels of serum LA, ALA, EPA, and DHA each showed robust inverse associations with the risk of all‐cause mortality, not just EPA and DHA (Zhang et al. 2021). In the NHANES analysis, only higher serum EPA (not DHA) correlated to lower cardiovascular disease (CVD) mortality risk. However, in a recent meta‐analysis of de novo data from 17 observational cohorts (O'Keefe et al. 2024), higher circulating DHA levels were associated with reduced all‐cause and specific cardiovascular and cancer mortality risks. As noted, dietary RBO significantly increases circulating EPA. In the NHANES analysis, only higher serum ALA correlated to lower cancer death risk—not LA, EPA, or DHA. RBO is a significant source of ALA, having about 75% of the ALA content of flaxseed oil with comparably low LA and a 4:1 omega‐3:6 ratio. The authors of the NHANES study concluded: “In consideration of associations between serum SDA and long‐chain n‐3 PUFAs together with lower intake of marine‐derived fish and environmental chemical pollutants, SDA‐rich oil may safely and expediently increase the concentration of serum EPA or other long‐chain n‐3 PUFAs to play their beneficial roles” (Zhang et al. 2021).

The lower levels of SDA in circulating cells compared to EPA or DHA should not be interpreted as a sign of lower intrinsic value epidemiologically. Instead, it may suggest that SDA has greater metabolic utility, contributing to a more diverse range of anti‐inflammatory oxylipins and influencing cytokine production. In this context, consuming only preformed EPA/DHA sources in some respects may deny or impede the body's biologically expected omega‐3 PUFA synthesis pathways. A more holistic view recognizes the role that adipose tissue fat reserves, high background omega‐6 linoleic acid (LA) from commodity seed oils in the Western diet, lifestyle choices, genetics, sex, and gut microbiome interactions all play in impacting DHA biosynthesis efficiency. Rising global EPA/DHA demand for aquaculture needs, rising ocean temperatures, and falling fat content in anchovies, sardines, mackerel, and so forth, all combine to put more pressure on the world's wild‐harvested omega‐3 oily fish populations. Terrestrially farmed SDA (and ALA) sources are alternatives and can be utilized alongside algal EPA/DHA sources in the diet to improve human EPA status and gain wider CVD prevention benefits sustainably.

While maintaining healthy DHA levels is essential for functional cell membranes and many key physiological processes, emerging evidence suggests that consuming high levels of preformed DHA, instead of following the human evolutionary aligned ALA‐SDA‐EPA‐DHA biosynthesis pathway, may not be physiologically necessary for many healthy adults (Burdge 2022). There are specific contexts like pregnancy, infancy, traumatic brain injury, and certain medical applications where improved DHA status is certainly warranted. This is not to make an argument for SDA's biological essentiality, rather that SDA‐rich dietary oils can play a useful role in improving EPA status sustainably and will side‐step now‐recognized omega‐3 metabolic pathway suppression/disruption effects associated with higher preformed DHA intakes. Furthermore, as previously discussed, there is recent evidence that high preformed DHA intake can inhibit the conversion of ALA to DHA in the liver of mice, as the body downregulates endogenous DHA biosynthesis in response to sufficient dietary DHA (Metherel, Valenzuela, et al. 2024). Similarly, the suppression of ALA to DHA conversion can occur when DHA is readily available from dietary sources, which may have implications for optimal omega‐3 PUFA intake, metabolism, and status (Burdge and Calder 2005).

A healthy Mediterranean‐style plant‐based diet providing sufficient ALA, alongside a rich SDA source like RBO or RBO with algal DHA, can be consumed in place of marine EPA/DHA sources to maintain consequential and physiologically relevant DHA levels in the body.

Furthermore, vegans, who consume no fish and form all their DHA from plant‐sourced omega‐3 PUFAs, are recognized for having consistently lower circulating EPA and DHA levels than omnivores (Menzel et al. 2022; García‐Maldonado et al. 2023), yet are generally recognized as having better overall health (Jabri et al. 2021; Agnoli et al. 2023), although that might not be the case for all possible long‐term outcomes (Katonova et al. 2022; Kraselnik 2024). Like omnivores, vegans have much higher circulating DHA than ALA or EPA levels, despite consuming little or no preformed DHA. This could indicate that vegans have higher tissue‐dependent EPA and DHA biosynthesis rates or greater EPA and DHA retention to maintain healthy omega‐3 PUFA‐mediated cell membrane functions. Consuming plant‐based dietary SDA sources like RBO can support EPA and DHA biosynthesis.

### 1.4 Recognizing SDA as an Effective, Sustainable Omega‐3 Source

Whether approaching omega‐3 PUFAs from cellular, key tissue, epidemiological, or eco‐sustainability standpoints, broadening dietary omega‐3 PUFA intakes to include SDA‐rich sources has rising scientific support. An “all hands on deck” approach is required to achieve improved omega‐3 PUFA status in children and adults and greater overall health and well‐being. RBO's balanced fatty acid composition, as a source of bioactive omega‐6 and omega‐3 PUFAs, coupled with its highest combined ALA + SDA content and its regeneratively grown, fully traceable supply chain, supports wider recommended use across a broad array of nutraceutical and food/beverage applications.

As people's cardiovascular, immune‐inflammatory, gut, skin, and mental health goals via omega‐3 PUFA nutrition increasingly conflict with unsustainable global demand including from aquaculture producers for marine omega‐3 sources, it is crucial that plant‐based omega‐3 dietary intakes are embraced broadly (Baker 2023) and with confidence that no inherent physiological trade‐offs in DHA biosynthesis or tissue maintenance will occur in doing so.

## Author Contributions

E.J.B. and G.C. collaborated in writing the initial draft. P.H. contributed substantial clinical insights. All authors contributed to and approved the final draft of the manuscript.

## Ethics Statement

The authors have nothing to report.

## Conflicts of Interest

E.J.B. collaborates with Natures Crops International. P.H. has no conflicts of interest to declare. G.C. is an employee of Natures Crops International, producers of RBO.

## Data Availability

No new data were generated or analyzed in this study.
